# Association between metabolic syndrome and osteoporosis in Taiwanese middle-aged and elderly participants

**DOI:** 10.1007/s11657-018-0467-z

**Published:** 2018-04-28

**Authors:** Hsin-Hui Lin, Chun-Yuan Huang, Lee-Ching Hwang

**Affiliations:** 1 The Department of Family Medicine, Mackay Memorial Hospital, No. 92, Sec. 2, Zhongshan N. Rd., Zhongshan Dist., 104 Taipei City, Taiwan, Republic of China; 2 The Department of Family Medicine, China Medical University Hospital Taipei Branch, Taipei, Taiwan; 3 The Department of Medicine, Mackay Medical College, New Taipei City, Taiwan

**Keywords:** Body mass index, Metabolic syndrome, Osteoporosis, Bone mineral density, Dual-energy X-ray absorptiometry

## Abstract

**Summary:**

This study examined the association between metabolic syndrome and osteoporosis among middle-aged and elderly Taiwanese participants. After controlling for body mass index, age, liver and renal functions, and nutrition and exercise statuses, we found no significant association between MS and osteoporosis in either gender.

**Purpose:**

The term metabolic syndrome (MS) encompasses different abnormalities with independent effects on bone metabolism, which has led to inconsistencies in the association between MS and osteoporosis. This study evaluated this association among middle-aged and elderly Taiwanese participants by adjusting relevant covariates.

**Methods:**

We enrolled 2007 participants (1045 men and 962 women) older than 50 years, who underwent a health examination at a preventive examination agency in urban Taiwan. We studied age, gender, diabetes mellitus and hypertension histories, smoking and exercise statuses, metabolic and nutrition indices, and liver and renal function profiles. We conducted multiple logistic regression analyses to examine the association between MS and osteoporosis by categorizing participants in terms of gender and body mass index (BMI).

**Results:**

Overall, men with osteoporosis were less likely to have MS, and displayed fewer MS components than men without osteoporosis; but we found no significant associations between MS, or its components, and osteoporosis in women. After forming two groups according to BMI and adjusting for covariates, we found no association between MS and osteoporosis in any group. Multiple logistic regression analysis revealed that regular exercise had a negative association with osteoporosis in the low BMI group for men (OR, 0.365; *p* = 0.008).

**Conclusions:**

After BMI stratification and adjustments for age, nutrition status, liver and renal functions, and exercise status, we found no significant association between MS and osteoporosis in either gender. Regular exercise may prevent osteoporosis, particularly in men with a lean body mass.

## Introduction

Osteoporosis is one of the most common metabolic diseases leading to the development of fractures and subsequent disability and causing morbidity and mortality in adults older than 50. The National Health and Nutrition Survey in Taiwan estimated the overall prevalence of osteoporosis at 12.3% from 2013 to 2015 [1]. And, some estimates predict a continued increase in its prevalence due to increasing aging populations [2, 3].

Metabolic syndrome (MS) is also a highly prevalent condition worldwide, including in Taiwan. Its global prevalence (based on the NCEP-ATP III criteria) varies from 8–24% in men and 7–46% in women [4]. A national survey in Taiwan set the local prevalence at 19.7% in 2007 [5]. MS combines metabolic alterations including abdominal obesity, altered glucose metabolism, dyslipidemia, and hypertension. This leads to higher morbidity and mortality rates in those affected, and to the development of cardiovascular diseases and diabetes mellitus [6, 7, 8]. The broadly documented vascular harm caused by it and recent studies has shown that MS may have a role in the development of osteoporosis [9–12].

MS components may affect bone metabolism through their independent mechanisms. For example, an increased abdominal circumference may increase cytokines and stimulate osteoclast differentiation. Decreased high-density lipoprotein cholesterol (HDL-C) levels may inhibit osteogenic activity in vascular cells and may regulate the differentiation of osteoblasts. An elevated blood pressure (BP) may stimulate urinary calcium excretion, and increased triglycerides (TG) and plasma glucose levels may also cause hypercalciuria. Additionally, the microvascular complications of diabetes mellitus may reduce blood flow to the bone [9, 13]. MS induces inflammation and calcium excretion, which can increase the risk of osteoporosis. It is also highly associated with obesity, a strong protective factor against osteoporosis due to the increased mechanical load on bones [14]. However, studies have provided inconsistent results on the association between MS and osteoporosis [15–19]. A negative association between MS and osteoporosis, or low bone mass density, was shown to be reversed after BMI adjustment [12].

Given the growing prevalence of both MS and osteoporosis, the doubts regarding their association and the lack of relevant information in Taiwan, our aim was to look for an association between MS and osteoporosis among Taiwanese adults older than 50 years of age by adjusting for related covariates.

## Methods

### Study population

The lowest age limit for diagnosing osteoporosis is 50 years. Therefore, we included participants who underwent a health test at a preventive examination agency in Taipei, Taiwan, and were older than 50. After excluding participants with abnormal thyroid-stimulating hormone or free T4 levels and those who regularly consumed alcoholic beverages, we enrolled 1045 men and 962 women for this cross-sectional study. The Institutional Review Board of Mackay Memorial Hospital, in Taiwan, approved this study (No. 12MMHIS092).

### Data collection

We used the MS definition of the Bureau of Health Promotion (Department of Health, Taiwan). Accordingly, MS was confirmed if 3 or more of the following conditions were present: (1) Waist circumference ≥ 80 cm in women or ≥ 90 cm in men. (2) High BP (systolic BP ≥ 130 mmHg or diastolic BP ≥ 85 mmHg) or hypertension history. (3) TG ≥ 150 mg/dL. (4) HDL-C < 50 mg/dL in women or < 40 mg/dL in men. (5) Fasting plasma glucose (FPG) ≥ 100 mg/dL or a diabetes mellitus history. The bone mineral density (BMD) in each participant was measured at the lumbar spine, total hip, and femoral neck using dual-energy X-ray absorptiometry (Lunar Prodigy Advance; GE Healthcare, Madison, WI, USA). Osteoporosis was defined as BMD values of 2.5 standard deviation or more below the mean value for young adults (*T* score ≤ 2.5) based on the lowest *T* score of measured skeletal site as proposed by the International Society for Clinical Densitometry (one diagnostic category) [20].

We also recorded other information, including age, gender, history of diabetes mellitus and hypertension, and smoking and exercise status. The BMI was calculated by dividing weight (kg) by height (m^2^). Waist circumferences were measured in the horizontal plane midway between the lowest rib and iliac crest. We obtained blood biochemical test results, including FPG, triglycerides, total cholesterol, HDL-C, low-density lipoprotein cholesterol (LDL-C), hemoglobin, albumin, aspartate aminotransferase (AST), alanine aminotransferase (ALT), and creatinine for each participant.

### Statistical analysis

Data are presented using frequencies and percentages for categorical variables. Chi square statistics were used to analyze categorical data, whereas the independent *t* test was used to compare continuous variables. We performed a multivariate logistic regression analysis with gender stratification and BMI grouping with adjustments for age, exercise status, creatinine, AST, and hemoglobin levels. A *p* < 0.05 was considered to be statistically significant. All statistical calculations were performed using the Statistical Package for the Social Sciences software for Windows version 22.0 (IBM, Armonk, NY, USA).

## Results

The participants’ ages ranged from 50 to 93 years, with an average of 58.9 years. In total, 62 men (5.9%) and 142 women (14.8%) were diagnosed with osteoporosis, and 428 men (41.0%) and 234 women (24.3%) were diagnosed with MS. The average *T* score was − 0.29 ± 1.48 for men and − 1.02 ± 1.41 for women. We found significant differences among the baseline characteristics between men and women (Table 1); therefore, we divided the study population according to gender. Table 2 shows the comparison between the participants with and without osteoporosis stratified by gender. Our results suggest that men with osteoporosis are less likely to practice regular exercise (*p* = 0.008) and suffer from hypertension (*p* = 0.011). They had lower BMIs (*p* < 0.001), and hemoglobin (*p* = 0.018), AST (*p* = 0.045), and creatinine (*p* = 0.040) levels. Their waist circumference (*p* < 0.001) was smaller, and their diastolic BP (*p* = 0.106) was lower than those in men without osteoporosis. Overall, they were less likely to meet the waist circumference (*p* = 0.010) and BP criteria for MS (*p* = 0.029) and were less likely to have MS (*p* = 0.025) or MS components (*p* = 0.035) than the men without osteoporosis. However, age, smoking status, diagnosis of diabetes mellitus, systolic BP, albumin, ALT, diagnosis of CKD, total cholesterol, TG, HDL-C, LDL-C, and FPG were not associated with the development or lack of osteoporosis in men.

**Table 1:** Baseline characteristics of study participants

	All (*n* = 2007)	Men (*n* = 1045)	Women (*n* = 962)	*p* value
Age (years)	58.9 ± 6.3	58.9 ± 6.3	58.9 ± 6.3	0.890
Current smoker (*n*, %)	205 (10.2%)	177 (16.9%)	28 (2.9%)	< 0.001
Regular exercise (*n*, %)	732 (36.5%)	402 (38.5%)	330 (34.3%)	0.053
Hypertension (*n*, %)	621 (30.9%)	377 (36.1%)	244 (25.4%)	< 0.001
Diabetes mellitus (*n*, %)	284 (14.2%)	165 (15.8%)	119 (12.4%)	0.028
Body weight (kg)	64.2 ± 11.4	70.8 ± 9.4	57.0 ± 8.7	< 0.001
Body mass index (kg/m^2^)	24.0 ± 3.2	24.7.5 ± 2.8	23.2 ± 3.4	< 0.001
≥ 27 (*n*, %)	307 (15.3%)	183 (17.5%)	124 (12.9%)	< 0.001
24–27 (*n*, %)	653 (32.5%)	437 (41.8%)	216 (22.5%)	
18.5–24 (*n*, %)	996 (49.6%)	413 (39.5%)	583 (60.6%)	
< 18.5 (*n*, %)	51 (2.5%)	12 (1.1%)	39 (4.1%)	
Waist circumference (cm)	83.7 ± 9.4	88.0 ± 7.9	79.1 ± 8.6	< 0.001
Systolic blood pressure (mmHg)	118.0 ± 15.9	120.5 ± 214.0	115.1 ± 17.2	< 0.001
Diastolic blood pressure (mmHg)	74.5 ± 10.0	77.0 ± 9.1	71.8 ± 10.1	< 0.001
Hemoglobin (mg/dL)	14.08 ± 1.48	14.97 ± 1.20	13.12 ± 1.09	< 0.001
Albumin (mg/dL)	4.29 ± 0.24	4.31 ± 0.24	4.28 ± 0.25	0.005
Aspartate aminotransferase (mg/dL)	24.7 ± 10.7	25.2 ± 10.2	24.2 ± 10.9	0.032
Alanine aminotransferase (mg/dL)	27.9 ± 20.2	30.6 ± 19.7	24.8 ± 19.8	< 0.001
Creatinine (mg/dL)	0.86 ± 0.19	0.97 ± 0.16	0.73 ± 0.13	< 0.001
Chronic kidney disease (eGFR, < 60) (*n*, %)	54 (2.7%)	30 (2.9%)	24 (2.5%)	0.603
Total cholesterol (mg/dL)	203.0 ± 35.3	196.6 ± 34.4	209.9 ± 35.0	< 0.001
Triglycerides (mg/dL)	118.1 ± 73.0	130.8 ± 80.1	104.9 ± 60.6	< 0.001
HDL-C (mg/dL)	59.0 ± 15.6	52.8 ± 12.9	65.7 ± 15.6	< 0.001
LDL-C (mg/dL)	120.9 ± 29.8	119.5 ± 29.6	122.5 ± 30.1	0.025
Fasting plasma glucose (mg/dL)	105.2 ± 20.2	107.9 ± 21.5	102.4 ± 17.9	< 0.001
Metabolic syndrome (*n*, %)	662 (33.0%)	428 (41.0%)	234 (24.3%)	< 0.001
Number of metabolic syndrome components	1.9 ± 1.4	2.3 ± 1.3	1.5 ± 1.3	< 0.001
1 (*n*, %)	459 (22.9%)	211 (20.2%)	248 (25.8%)	< 0.001
2 (*n*, %)	510 (25.4%)	307 (29.4%)	203 (21.1%)	
3 (*n*, %)	381 (19.0%)	224 (21.4%)	157 (16.3%)	
4 (*n*, %)	208 (10.4%)	152 (14.5%)	56 (5.8%)	
5 (*n*, %)	73 (3.6%)	52 (5.0%)	21 (2.2%)	
Bone mineral density, *T* score	− 0.63 ± 1.49	− 0.29 ± 1.48	− 1.02 ± 1.41	< 0.001
Osteopenia (*n*, %)	682 (34.0%)	296 (28.3%)	385 (40.0%)	< 0.001
Osteoporosis (*n*, %)	204 (10.2%)	62 (5.9%)	142 (14.8%)	< 0.001

*HDL-C* high-density lipoprotein cholesterol, *LDL-C* low-density lipoprotein cholesterol

**Table 2:** Comparison between the participants with and without osteoporosis stratified by gender

Variants	Men			Women		
	No osteoporosis (*n* = 983)	Osteoporosis (*n* = 62)	*p* value	No osteoporosis (*n* = 820)	Osteoporosis (*n* = 142)	*p* value
Age (years)	58.8 ± 6.2	59.6 ± 7.9	0.430	58.2 ± 5.9	62.7 ± 6.9	< 0.001
Current smoker (*n*, %)	164 (16.7%)	13 (21.0%)	0.383	23 (2.8%)	5 (3.5%)	0.639
Regular exercise (*n*, %)	388 (39.5%)	14 (22.6%)	0.008	275 (33.5%)	55 (36.2%)	0.229
Hypertension (*n*, %)	364 (37.0%)	13 (21.0%)	0.011	198 (24.1%)	46 (32.4%)	0.037
Diabetes mellitus (*n*, %)	154 (15.7%)	11 (17.7%)	0.664	88 (10.7%)	31 (21.8%)	< 0.001
Body weight (kg)	71.3 ± 9.3	64.3 ± 10.0	< 0.001	57.6 ± 8.7	53.3 ± 7.4	< 0.001
Body mass index (kg/m^2^)	24.75 ± 2.72	23.05 ± 2.98	< 0.001	23.39 ± 3.46	22.36 ± 3.06	< 0.001
≥ 27 (*n*, %)	177 (18.0%)	6 (9.7%)	<0.001	109 (13.2%)	15 (10.6%)	0.043
24–27 (*n*, %)	423 (43.0%)	14 (22.6%)		193 (23.5%)	23 (16.2%)	
18.5–24 (*n*, %)	374 (38.0%)	39 (62.9%)		489 (59.6%)	94 (66.2%)	
< 18.5 (*n*, %)	9 (1.0%)	3 (4.8%)		29 (3.5%)	10 (7.0%)	
Waist circumference (cm)	88.2 ± 7.8	83.6 ± 8.0	< 0.001	79.3 ± 8.6	78.3 ± 9.1	0.232
Systolic blood pressure (mmHg)	120.7 ± 14.1	118.0 ± 12.7	0.106	114.8 ± 17.0	117.8 ± 18.8	0.059
Diastolic blood pressure (mmHg)	77.2 ± 9.1	74.6 ± 9.0	0.030	71.9 ± 10.1	71.1 ± 10.1	0.379
Hemoglobin (mg/dL)	15.0 ± 1.2	14.7 ± 1.0	0.018	13.1 ± 1.1	13.1 ± 1.0	0.759
Albumin (mg/dL)	4.31 ± 0.23	4.31 ± 0.25	0.914	4.28 ± 0.25	4.26 ± 0.23	0.316
Aspartate aminotransferase (mg/dL)	25.3 ± 10.4	23.4 ± 6.8	0.045	24.0 ± 10.1	25.7 ± 15.7	0.093
Alanine aminotransferase (mg/dL)	30.8 ± 20.4	27.4 ± 11.5	0.053	25.0 ± 20.1	24.5 ± 19.8	0.810
Creatinine (mg/dL)	0.98 ± 0.16	0.93 ± 0.16	0.040	0.73 ± 0.14	0.72 ± 0.11	0.530
Chronic kidney disease (eGFR < 60) (*n*, %)	28 (2.8%)	2 (3.2%)	0.863	20 (2.4%)	4 (2.8%)	0.790
Total cholesterol (mg/dL)	196.5 ± 34.3	199.3 ± 35.6	0.540	209.6 ± 35.0	211.6 ± 35.0	0.517
Triglycerides (mg/dL)	130.5 ± 78.5	132.0 ± 111.1	0.914	105.0 ± 62.4	101.6 ± 49.6	0.474
HDL-C (mg/dL)	52.8 ± 12.7	54.1 ± 15.0	0.485	65.4 ± 15.6	67.1 ± 15.5	0.224
LDL-C (mg/dL)	119.5 ± 29.5	120.2 ± 31.1	0.855	122.3 ± 30.3	123.4 ± 29.0	0.689
Fasting plasma glucose (mg/dL)	108.0 ± 21.9	106.3 ± 16.7	0.458	101.8 ± 17.4	106.1 ± 21.1	0.009
Waist circumference ≥ 90 cm in men, ≥ 80 cm in women (*n*, %)	400 (40.7%)	15 (24.2%)	0.010	357 (43.5%)	57 (40.1%)	0.451
SBP ≥ 130 or DBP ≥ 85 or hypertension history (*n*, %)	457 (46.5%)	20 (32.3%)	0.029	243 (29.6%)	57 (40.1%)	0.013
Triglycerides ≥ 150 mg/dL	275 (28.0%)	16 (25.8%)	0.712	133 (16.2%)	22 (15.5%)	0.828
HDL-C < 40 mg/dL in men, 50 mg/dL in women (*n*, %)	457 (46.5%)	29 (46.8%)	0.965	117 (14.3%)	17 (12.0%)	0.466
Fasting plasma glucose, ≥ 100 mg/dL or diabetes history (*n*, %)	659 (67.0%)	37 (59.7%)	0.233	377 (46.0%)	74 (52.1%)	0.176
Metabolic syndrome (*n*, %)	411 (41.8%)	17 (27.4%)	0.025	198 (24.1%)	36 (25.4%)	0.757
Number of metabolic syndrome components	2.3 ± 1.3	1.9 ± 1.4	0.035	1.5 ± 1.3	1.6 ± 1.3	0.388
1 (*n*, %)	197 (20.0%)	14 (22.6%)	0.023	204 (24.9%)	44 (31.0%)	0.530
2 (*n*, %)	288 (29.3%)	19 (30.6%)		173 (21.1%)	30 (21.1%)	
3 (*n*, %)	219 (22.3%)	5 (8.1%)		133 (16.2%)	24 (16.9%)	
4 (*n*, %)	142 (14.4%)	10 (16.1%)		47 (5.7%)	9 (6.3%)	
5 (*n*, %)	50 (5.1%)	2 (3.2%)		18 (2.2%)	3 (2.1%)	

*eGFR* estimated glomerular filtration rate, *HDL-C* high-density lipoprotein cholesterol, *LDL-C* low-density lipoprotein cholesterol, *SBP* systolic blood pressure, *DBP* diastolic blood pressure

Women with osteoporosis were older (*p* < 0.001), were more likely to have hypertension (*p* = 0.037) or diabetes mellitus (*p* < 0.001), had lower BMIs (*p* < 0.001) and higher FPG (*p* = 0.009) levels, and were more likely to meet the BP criteria for MS (*p* = 0.013) compared with those without osteoporosis. However, no differences were apparent after examining other variables, including smoking status, exercise status, waist circumference, systolic BP, diastolic BP, hemoglobin, albumin, AST, ALT, creatinine, diagnosis of CKD, total cholesterol, TG, HDL-C, LDL-C, diagnosis of metabolic syndrome (*p* = 0.757), or the number of MS components (*p* = 0.388) in women.

After dividing the participants into two groups according to their BMI levels (using 24 kg/m^2^ as a cut-off value) and adjusting for age, exercise status, creatinine, AST, and hemoglobin levels, we performed multiple logistic regression analysis for odds ratio of associated factors for osteoporosis in men and women (Tables 3 and 4). The exercise status was negatively associated with osteoporosis in men of the low BMI group (OR, 0.365; 95% CI, 0.174–0.765; *p* = 0.008). We found age to be positively associated with osteoporosis in the men of the low BMI group (OR, 1.054; 95% CI, 1.004–1.106; *p* = 0.033), and in the women of both BMI groups (OR, 1.133; 95% CI, 1.091–1.176; *p* < 0.001 in the low BMI group and OR, 1.115; 95% CI, 1.061–1.172; *p* < 0.001 in the high BMI group). We found no association between osteoporosis and MS in the other BMI or gender groups [(OR, 0.579; 95% CI, 0.213–1.575; *p* = 0.284 in men with low BMI); (OR, 0.986; 95% CI, 0.390–2.496; *p* = 0.977 in men with high BMI); (OR, 0.965; 95% CI, 0.457–2.038; *p* = 0.901 in women with low BMI); and (OR, 1.060; 95% CI, 0.493–2.281; *p* = 0.881 in women with high BMI)]. The MS components and its numbers were also not significantly associated with osteoporosis.

**Table 3:** Associated factors of osteoporosis in men with calculation of adjusted odds ratio using multiple logistic regression analysis

Variants	Osteoporosis in BMI < 24 group			Osteoporosis in BMI ≥ 24 group		
	Odds ratio	95% CI	*p* value	Odds ratio	95% CI	*p* value
Regular exercise	0.365	0.174–0.765	0.008	0.359	0.102–1.162	0.110
Age (years)	1.054	1.004–1.106	0.033	0.959	0.876–1.050	0.368
Hemoglobin	0.961	0.714–1.292	0.790	0.769	0.532–1.111	0.162
Aspartate aminotransferase	0.954	0.899–1.012	0.118	1.006	0.974–1.039	0.710
Creatinine	0.254	0.032–2.482	0.254	0.011	0.000–0.514	0.021*
Metabolic syndrome^a^	0.579	0.213–1.575	0.284	0.986	0.390–2.496	0.977
Number of MS components^a^	0.080	0.710–1.298	0.416	0.953	0.645–1.408	0.810
Waist circumference ≥ 90cm^a^	0.870	0.184–4.103	0.860	1.047	0.402–2.726	0.924
Triglycerides ≥ 150^a^	0.751	0.296–1.904	0.546	1.724	0.690–4.310	0.244
HDL-C < 40 mg/dL^a^	1.422	0.723–2.796	0.307	0.912	0.361–2.307	0.846
SBP ≥ 130 or DPB ≥ 85 or hypertension history^a^	0.892	0.439–1.873	0.752	0.425	0.157–1.147	0.091
Fasting plasma glucose ≥ 100 mg/dL^a^	0.785	0.408–1.510	0.468	1.079	0.377–3.088	0.888

^a^Adjusted for age, aspartate aminotransferase, creatinine, hemoglobin, and exercise status. *MS* metabolic syndrome, *HDL-C* high-density lipoprotein cholesterol, *SBP* systolic blood pressure, *DBP* diastolic blood pressure

**Table 4:** Associated factors of osteoporosis in women with calculation of adjusted odds ratio (OR) using multiple logistic regression analysis

Variants	Osteoporosis in BMI < 24 group			Osteoporosis in BMI ≥ 24 group		
	Odds ratio	95% CI	*p* value	Odds ratio	95% CI	*p* value
Regular exercise	0.999	0.623–1.580	0.998	0.872	0.398–1.910	0.733
Age (years)	1.133	1.091–1.176	<0.001	1.115	1.061–1.172	< 0.001
Hemoglobin	1.079	0.868–1.341	0.496	0.770	0.557–1.065	0.115
Aspartate aminotransferase	1.012	0.992–1.031	0.244	1.001	0.970–1.034	0.933
Creatinine	0.306	0.032–2.887	0.301	0.151	0.008–2.784	0.204
Metabolic syndrome^a^	0.965	0.457–2.038	0.425	1.060	0.493–2.281	0.881
Number of MS components^a^	1.073	0.872–1.320	0.508	1.050	0.754–1.462	0.774
Waist circumference≥80cm^a^	1.098	0.626–1.926	0.745	0.585	0.201–1.705	0.326
Triglycerides ≥ 150^a^	1.031	0.498–2.135	0.934	1.275	0.569–2.855	0.555
HDL-C < 50 mg/dL^a^	0.759	0.341–1.688	0.498	1.417	0.581–3.456	0.444
SBP ≥ 130 or DPB ≥ 85 or hypertension history^a^	0.883	0.397–1.962	0.759	1.235	0.733–2.080	0.427
Fasting plasma glucose ≥ 100 mg/dL^a^	1.221	0.776–1.921	0.388	1.189	0.532–2.660	0.673

^a^Adjusted for age, aspartate aminotransferase, creatinine, hemoglobin, and exercise status. *MS* metabolic syndrome, *HDL-C* high-density lipoprotein cholesterol, *SBP* systolic blood pressure, *DBP* diastolic blood pressure

## Discussion

The prevalence of MS in our study was found to be 33.0% (41.0% in men and 24.3% in women), which is higher than that reported for the general population of Taiwan (19.7%) [5] but is closer to that for the population older than 50 in a Taiwanese survey in 2017 (34.2%) [17]. The prevalence of osteoporosis was found to be 10.2% (5.9% in men and 14.8% in women), which is lower than that in the general population of Taiwan (12.3%) [1]. Our study participants came from an urban self-paid health exam center and were supposed to have a higher social economic status with better nutrition, medical knowledge, and self-health awareness than the rest of the Taiwanese population. This may justify the differences observed.

The studies on the relationship between MS and BMD have yielded contradictory results. El Maghraoui et al. showed that women with MS had higher BMDs at the hip and spine [16]. A meta-analysis by Xue et al. reported that MS may have a beneficial influence on BMD [21]. On the other hand, Von Muhlen et al. reported that MS is associated with lower BMDs after adjusting for age and BMI [12]. A meta-analysis by Sugimoto et al. also suggested that MS is associated with bone loss in Asian men [11]. Jang et al. showed that after adjusting for BMI and age, MS was not associated with higher BMD in Korean women [22]. Heidary et al. revealed similar results in elderly Iranian males [23]. Further, Loke et al. reported that MS was positively associated with BMD in men; however, the association was negative in women, revealing a gender difference [17]. In addition to the inconsistent results, most of the sample sizes in these studies are insufficient. Furthermore, some analyses were not adjusted for BMI or other covariates.

In our study, we found that osteoporosis is negatively associated with MS and the number of MS components in men before adjustments, but not in women. Among all these components, a high BP and increased waist circumference were associated with less osteoporosis in men, whereas increased fasting plasma sugar and high BP were associated with more osteoporosis in women. After performing a stratified analysis of BMI and adjusting for age, nutrition status, liver and renal functions, and exercise status, these associations disappeared, and there were no significant associations between MS and osteoporosis in either gender. Studies have suggested that obesity acts as a strong protective factor against bone loss in older people [14, 24]. Obesity increases the load on bones, and is associated with insulin resistance resulting in hyperinsulinemia, which leads to increased bone formation [25]. Therefore, we assume that this association before adjusting for covariates may have contributed to the high association with obesity of these covariates.

Other than age, only the exercise status remained significantly associated with osteoporosis after adjustments. Our results showed that regular exercise decreased the risk of osteoporosis (OR, 0.365; *p* = 0.008) in men of the low BMI group. This result is compatible with studies on the major osteoporosis risk factors, which include aging, calcium deficiency, vitamin D deficiency, genetic factors, physical inactivity, estrogen deficiency, and history of fractures [26, 27]. Lean men should be particularly encouraged to exercise to overcome the harmful effects of low BMI on bones.

We are aware of the limitations of cross-sectional studies like this one, and although the key strength of our study is its large population sample (larger than those in most previous studies), a large-scale prospective study is still required. Finally, considering that our study population included self-referred participants in a health promotion center, it may not be representative of the general population. In this article, we examined the effects of cardiometabolic factors on osteoporosis which was defined as a *T* score of − 2.5 standard deviation below BMD of a healthy young person of the same sex. The association between MS and BMD value was not analyzed since there is no original BMD value. Although the relationship between them needs more clarification, the *T* score has traditionally been used as a BMD criterion by researchers.

In conclusion, after performing a stratified analysis of BMI and adjusting for age, nutrition status, liver and renal functions, and exercise status, our statistical analysis did not find any significant associations between MS and osteoporosis in either gender. The negative association between MS and osteoporosis in men may be explained by the protective effects of higher BMI in participants with MS. However, well designed, large, and randomized trials and pathophysiological studies are needed to validate our results. Regular exercise may protect those with a lean body mass from osteoporosis, particularly if they are men.
